# Clinical significance of serum brain-derived neurotrophic factor and serotonin levels in children with tic disorders

**DOI:** 10.3389/fped.2026.1826133

**Published:** 2026-05-29

**Authors:** Xiaoxia Zhang, Na Chen, Xindong Zhu, Chen Wang

**Affiliations:** 1 Department of Pediatrics, Tongde Hospital of Zhejiang Province, Hangzhou, China; 2 Department of Pediatrics, The Fourth School of Clinical Medicine, Zhejiang Chinese Medical University (Hangzhou First People’s Hospital), Hangzhou, China

**Keywords:** 5-HT, brain-derived neurotrophic factor, disease severity, tic disorders, Tourette syndrome

## Abstract

**Objective:**

Tic disorders (TD) are diagnosed mainly through clinical observation, and accessible peripheral biomarkers that reflect neurobiological alterations remain limited. Brain-derived neurotrophic factor (BDNF) and serotonin (5-HT) are biologically relevant to synaptic plasticity and neurochemical regulation. This study aimed to investigate serum BDNF and 5-HT levels in children with TD and to explore their associations with disease severity and clinical subtypes.

**Methods:**

A total of 125 children with TD and 100 age- and sex-matched healthy controls were enrolled. Serum BDNF and 5-HT concentrations were measured using enzyme-linked immunosorbent assay (ELISA). TD patients were further classified into mild and moderate-to-severe groups according to the Yale Global Tic Severity Scale. Biomarker differences across severity strata and TD subtypes were analyzed, and diagnostic performance was assessed using receiver operating characteristic (ROC) curves and logistic regression.

**Results:**

Serum BDNF levels were significantly lower in TD patients than in healthy controls and declined further in moderate-to-severe cases. In contrast, serum 5-HT levels did not differ between mild TD and HC but were significantly reduced in moderate-to-severe TD compared with mild cases. ROC analysis showed that BDNF (AUC 0.705, 95% CI 0.637–0.772) had modest discriminative ability for identifying TD and both BDNF (AUC 0.760, 95% CI 0.678–0.842) and 5-HT (AUC 0.652, 95% CI 0.555–0.749) were useful in distinguishing disease severity. Logistic regression identified sleep disturbances, family history of TD, and decreased BDNF were independently associated with TD.

**Conclusion:**

Reduced serum BDNF was associated with disease presence and severity, whereas decreased 5-HT was mainly related to greater symptom severity. Given the modest ROC performance, serum BDNF and 5-HT may provide auxiliary biological information for pediatric tic disorders, but their clinical application requires validation in larger multicenter cohorts.

## Introduction

Tic disorders begin in childhood and are characterized by sudden, rapid, recurrent, and nonrhythmic motor or vocal tics. The current diagnosis is still based on the DSM-5-TR criteria, which take into account the course of the disease, age of onset, and exclusion conditions (1, 2). Epidemiological studies estimate that the prevalence of Tourette syndrome among children and adolescents is approximately 0.3%–0.9%, with a significantly higher prevalence in boys than in girls (3, 4). Tic disorders are often comorbid with neuropsychiatric conditions such as attention-deficit/hyperactivity disorder (ADHD) and obsessive–compulsive disorder (OCD), suggesting that their pathophysiology involves a broader imbalance in neural networks (5). Previous studies have supported that genetic susceptibility and adverse perinatal exposures—such as maternal smoking, premature birth, or low birth weight—may increase the risk of onset (6). More recently, a 2D:4D digit ratio study further suggested that prenatal hormonal exposure may be associated with tic disorder susceptibility, supporting a developmental endophenotype perspective for TD (7). In addition, abnormalities in immune and inflammatory pathways have been reported in tic disorders, suggesting that neuroimmune mechanisms may contribute to disease susceptibility in some patients (8).

From this developmental perspective, peripheral biomarkers related to neuroplasticity and neurotransmitter regulation may help characterize biological heterogeneity in pediatric TD. BDNF supports neuronal survival and synaptic plasticity within fronto-striatal circuits that are central to tic pathophysiology (9, 10). BDNF-dependent modulation of corticostriatal long-term plasticity provides a mechanistic link to motor control and inhibitory regulation (11). Given this biological plausibility, pediatric serum work specifically addressing BDNF in tic disorders remains limited in the literature (12). The serotonin (5-HT) system contributes to impulsivity and repetitive behaviors that accompany tics (13). Human molecular imaging demonstrates altered serotonin transporter binding in Tourette syndrome (14). In pediatric samples, a pilot case–control study reported group differences in plasma and urine neurotransmitters including 5-HT between children with tic disorders and healthy controls, yet the analysis did not stratify by comorbidity or symptom severity (15). Peripheral 5-HT measures show heterogeneous results across reports, underscoring the need for targeted pediatric studies with uniform methods (16).

Previous evidence on peripheral BDNF and 5-HT in pediatric tic disorders remains limited, and few studies have examined these markers in relation to tic severity and clinical subtypes. The present study aimed to determine whether serum BDNF and 5-HT concentrations differed between pediatric tic disorder patients and healthy controls and to clarify their associations with tic severity assessed by the Yale Global Tic Severity Scale. Stratified analyses according to comorbidity status and tic severity were designed to identify subgroup-specific patterns and reduce potential confounding. By integrating these parameters, this work sought to provide evidence supporting the clinical utility of serum BDNF and 5-HT as accessible biomarkers for disease assessment and individualized management in children with tic disorders.

## Methods

### Study population

This cross-sectional observational study enrolled 125 children diagnosed with TD who were admitted to or visited our hospital between January 2024 and January 2025. The diagnosis of tic disorders was established by experienced pediatric neurologists in accordance with the criteria of the Diagnostic and Statistical Manual of Mental Disorders, Fifth Edition, Text Revision (DSM-5-TR) (17). All participants met the diagnostic criteria and had a disease duration of at least twelve months prior to enrollment. The inclusion criteria were as follows: children aged between six and sixteen years, confirmed diagnosis of TD or persistent (chronic) motor or vocal tic disorder according to DSM-5-TR, and the ability to cooperate with clinical assessment and venous blood sampling. Participants were required to have at least one parent or legal guardian capable of understanding the study procedures and providing written informed consent. The exclusion criteria included children diagnosed with transient tic disorder, those with comorbid autoimmune diseases, and those presenting with acute febrile illness or suspected infection within the preceding two weeks. Patients with known major neurological or metabolic disorders, including epilepsy, a history of severe traumatic brain injury, or neurodegenerative disease, were excluded. Children who had received systemic corticosteroids or other immunomodulatory therapies within four weeks prior to enrollment, or who were unable to provide assent, were also excluded.

In addition, 100 age- and sex-matched healthy children who underwent routine health examinations at our hospital during the same period were recruited as controls. These individuals had no history of TD or other major neurological or psychiatric conditions and had not experienced acute infection within the previous two weeks. This study was conducted in accordance with the ethical principles of the Declaration of Helsinki and approved by the Institutional Review Board of our hospital (Approval No. LC2024-007). Written informed consent was obtained from the parents or legal guardians of all participants, and assent was obtained from children where appropriate.

### Yale global tic severity scale (YGTSS)

Tic severity was evaluated using the YGTSS, a clinician-administered instrument developed by Leckman et al. The YGTSS consists of three components. The first component separately rates motor tics and phonic tics across five dimensions: number, frequency, intensity, complexity, and interference. The second section quantifies tic severity by summing scores for these domains, yielding independent motor and vocal tic subscales, each ranging from 0 to 25, with a combined maximum total score of 50. The third section, known as the Impairment Score, evaluates the overall impact of tics on daily functioning and psychosocial well-being. In the present study, analysis was restricted to the second section of the YGTSS, which reflects the core severity of motor and vocal tics. Based on the total tic severity score, participants were categorized into two groups: mild (0–20) and moderate-to-severe (21–50) (18, 19).

### Blood sample collection and biomarker measurement

Venous blood samples were collected from all participants between 08:00 and 10:00 a.m. after an overnight fast of at least 8 h to minimize the effects of circadian and dietary variability. For patients with tic disorders, samples were obtained prior to any pharmacological treatment or behavioral intervention on the day of clinical assessment. All specimens were collected into sterile serum-separating tubes, allowed to clot at room temperature for 30 min, and centrifuged at 3,000 × g for 10 min at 4 °C. The supernatant serum was carefully aliquoted and stored at −80 °C until biochemical analysis.

Serum BDNF levels were measured using a commercial enzyme-linked immunosorbent assay (ELISA) kit (Catalog No. MBS039474, MyBioSource, San Diego, CA, USA) with a detection range of 62.5–2,000 pg/mL. The intra-assay coefficient of variation (CV) was≤15%, and the inter-assay CV was≤15%, according to the manufacturer's specifications. Serum 5-HT concentrations were determined using an ELISA kit (Catalog No. MBS263185, MyBioSource, San Diego, CA, USA) with a detection range of 1.56–100 ng/mL. The intra-assay precision was≤8%, and the inter-assay precision was≤12%. All measurements were performed in duplicate according to the manufacturers’ instructions, and the mean values were used for statistical analysis.

### Clinical data collection

Clinical information was obtained from all participants using a standardized data collection form. Variables collected included chronological age, sex, body mass index (BMI), platelet count, tic type (Tourette syndrome, chronic motor/vocal tic disorder), duration of illness, age at tic onset, premature birth status, history of febrile convulsion, allergy, asthma, sleep disturbances, comorbid attention-deficit/hyperactivity disorder (ADHD) or obsessive-compulsive disorder (OCD), and family history of TD. Previous treatment history for tic symptoms before enrollment was also recorded.

Comorbid ADHD and OCD were diagnosed by qualified child psychiatrists according to the DSM-5-TR (17).

### Statistical analysis

All statistical analyses were performed using SPSS version 26.0 (IBM Corp., Armonk, NY, USA). Data distribution was examined using the Shapiro–Wilk test. Normally distributed continuous variables were expressed as mean ± standard deviation and compared using the independent-samples t test. Non-normally distributed variables were presented as median (interquartile range) and compared using the Mann–Whitney U test. Categorical variables were expressed as frequencies and percentages and compared using the chi-square test. Spearman correlation analysis was conducted to assess the relationships between serum BDNF, 5-HT levels, and YGTSS. Receiver operating characteristic (ROC) curve analysis was performed to evaluate the diagnostic performance of serum BDNF and 5-HT in identifying children with TD and those with moderate-to-severe tic severity. Binary logistic regression analysis was further applied to determine independent risk factors associated with the presence of TD. Variables were selected for the multivariable logistic regression model based on clinical relevance, biological plausibility, and potential confounding effects. The final model included age, sex, BMI, premature birth, sleep disturbances, family history of TD, PLT, serum BDNF, and serum 5-HT. Model calibration was evaluated using the Hosmer–Lemeshow goodness-of-fit test, and multicollinearity was assessed using variance inflation factors (VIFs). A *p* value < 0.05 was considered statistically significant.

## Results

### Baseline clinical characteristics

This cross-sectional observational study enrolled 125 children diagnosed with TD (TD group) and 100 healthy controls (HC group). In the TD group, the median duration of illness was 3 (1–4) years, and the median age at tic onset was 7 (5–8) years. The median YGTSS total score was 20 (13–30). Comorbid ADHD was present in 50.4% of TD patients, and comorbid OCD in 19.2%. Regarding tic subtypes, 66.4% were diagnosed with Tourette syndrome and 33.6% with chronic motor/vocal tic disorder. When comparing demographic and clinical characteristics between the two groups, there were no significant differences in age, sex, or BMI. However, the TD group showed significantly higher proportions of premature birth, sleep disturbances, and a family history of tic disorders (*p* < 0.05), whereas all other parameters were comparable between groups (Table 1).

**Table 1:** Baseline clinical characteristics of TD patients and healthy controls.

Variable	TD, *n*=125	HC control, *n*=100	*p*
Age, y	12 (7–15)	11 (7.25–15)	0.579
Sex, boy (%)	69 (55.2)	58 (58.0)	0.674
BMI	18.23 (16.69–19.69)	18.79 (15.93–20.21)	0.409
Platelet, × 10⁹/L	303 (269.0–340.5)	300 (250.0–328.8)	0.234
Premature birth, n (%)	18 (14.4)	6 (6.0)	0.043
History of febrile convulsion, n (%)	9 (7.2)	6 (6.0)	0.720
Allergy, n (%)	31 (24.8)	23 (23.0)	0.753
Asthma, n (%)	11 (8.8)	7 (7.0)	0.621
Sleep disturbances, n (%)	66 (52.8)	23 (23.0)	<0.001
Comorbid ADHD, n (%)	63 (50.4)		
Comorbid OCD, n (%)	24 (19.2)		
Family history of TD, n (%)	19 (15.2)	4 (4.0)	0.006
Tic subtype			
Tourette syndrome, n (%)	83 (66.4)		
Chronic motor/vocal tic disorder, n (%)	42 (33.6)		
Duration of illness, y	3 (1–4)		
Age at tic onset, y	7 (5–8)		
YGTSS	20 (13–30)		
Previously treated	82 (65.6)		
treatment-naïve	43 (34.4)		

### Serum BDNF and 5-HT levels in children with TD

**Figure 1:**
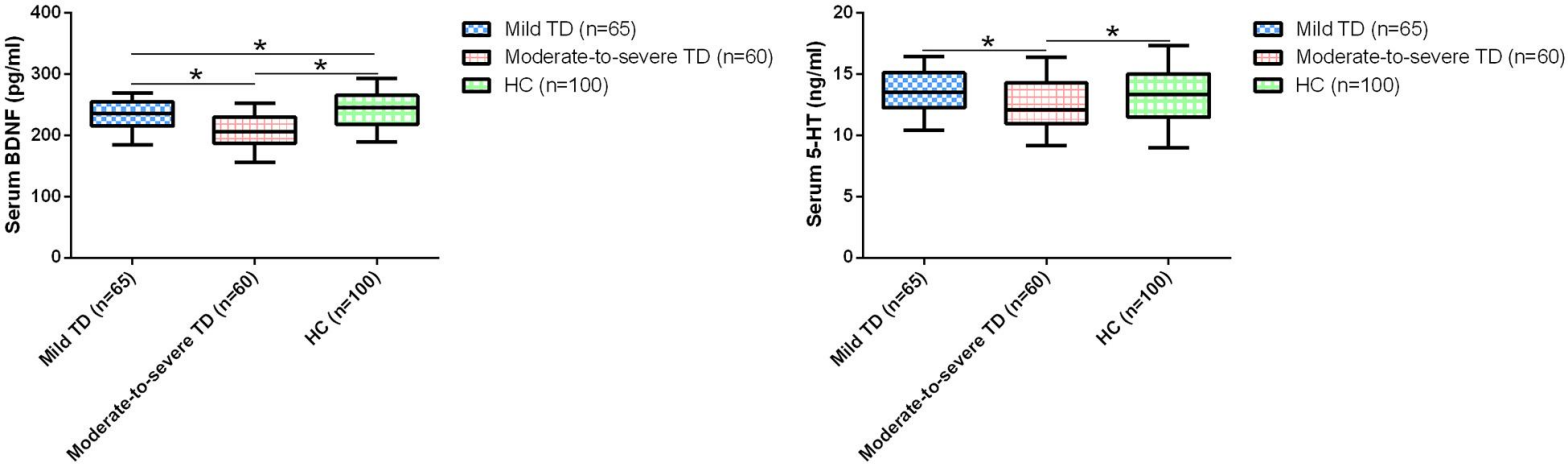
Serum BDNF and 5-HT levels in children with mild TD, moderate-to-severe TD, and healthy controls. BDNF, brain-derived neurotrophic factor; 5-HT, serotonin; TD, tic disorders; HC, healthy controls.

Serum BDNF and 5-HT levels were compared among children with mild TD (*n* = 65), moderate-to-severe TD (*n* = 60), and healthy controls (*n* = 100). Serum BDNF levels differed significantly among the three groups (Figure 1, *p* < 0.05), with both mild and moderate-to-severe TD groups showing lower serum levels than HC, and the lowest serum levels observed in the moderate-to-severe group (*p* < 0.05). In contrast, serum 5-HT levels were significantly lower in children with moderate-to-severe TD compared with both mild TD and HC (*p* < 0.05), whereas no significant difference was found between mild TD and healthy controls (*p* > 0.05).

**Figure 2:**
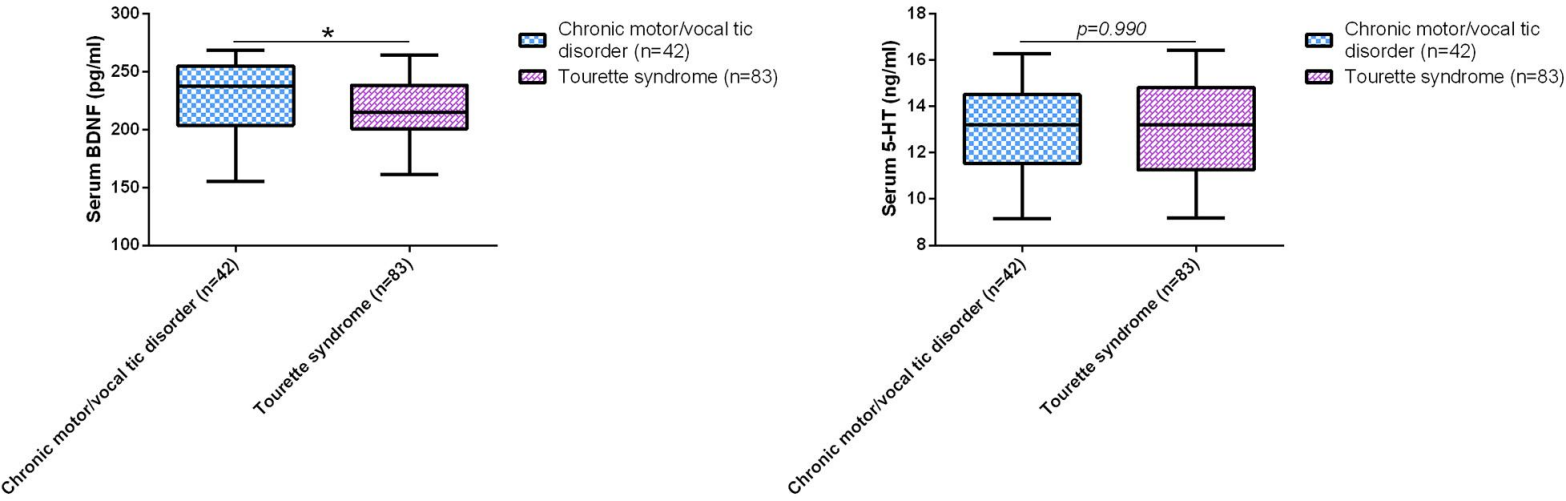
Comparison of serum BDNF and 5-HT levels between Tourette syndrome and chronic motor/vocal tic disorder. BDNF, brain-derived neurotrophic factor; 5-HT, serotonin.

When stratified by subtype, patients with Tourette syndrome (*n* = 83) exhibited significantly lower serum BDNF levels than those with chronic motor/vocal tic disorder (*n* = 42, Figure 2, *p* < 0.05), whereas no significant difference was observed in 5-HT levels between the two subtypes (*p* > 0.05).

**Figure 3:**
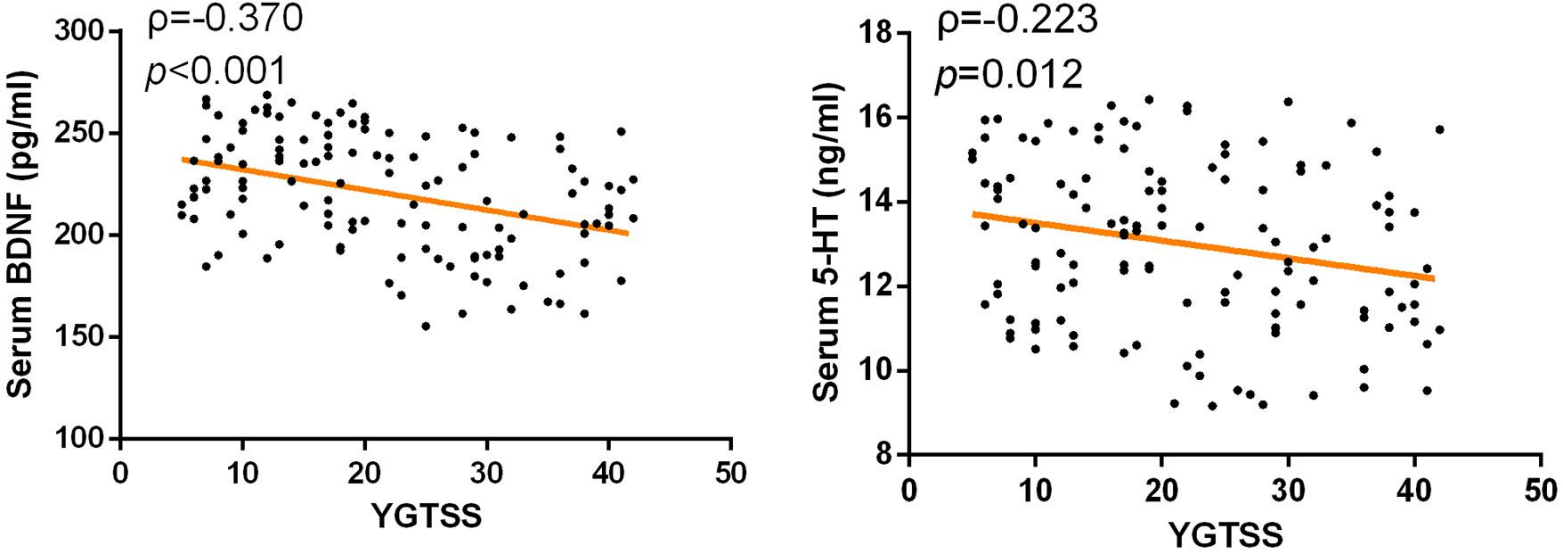
Scatter plots showing correlations between serum BDNF or 5-HT levels and YGTSS total scores in children with TD. BDNF, brain-derived neurotrophic factor; 5-HT, serotonin; YGTSS, Yale Global Tic Severity Scale; TD, tic disorders.

Spearman correlation analysis showed that serum BDNF levels had a weak negative correlation with total YGTSS scores (*ρ*=−0.370, *p* < 0.001), and serum 5-HT levels also exhibited a weaker but significant negative correlation (*ρ*=−0.223, *p* = 0.012) (Figure 3).

### Serum BDNF and 5-HT in TD subgroups with and without comorbidity

**Figure 4:**
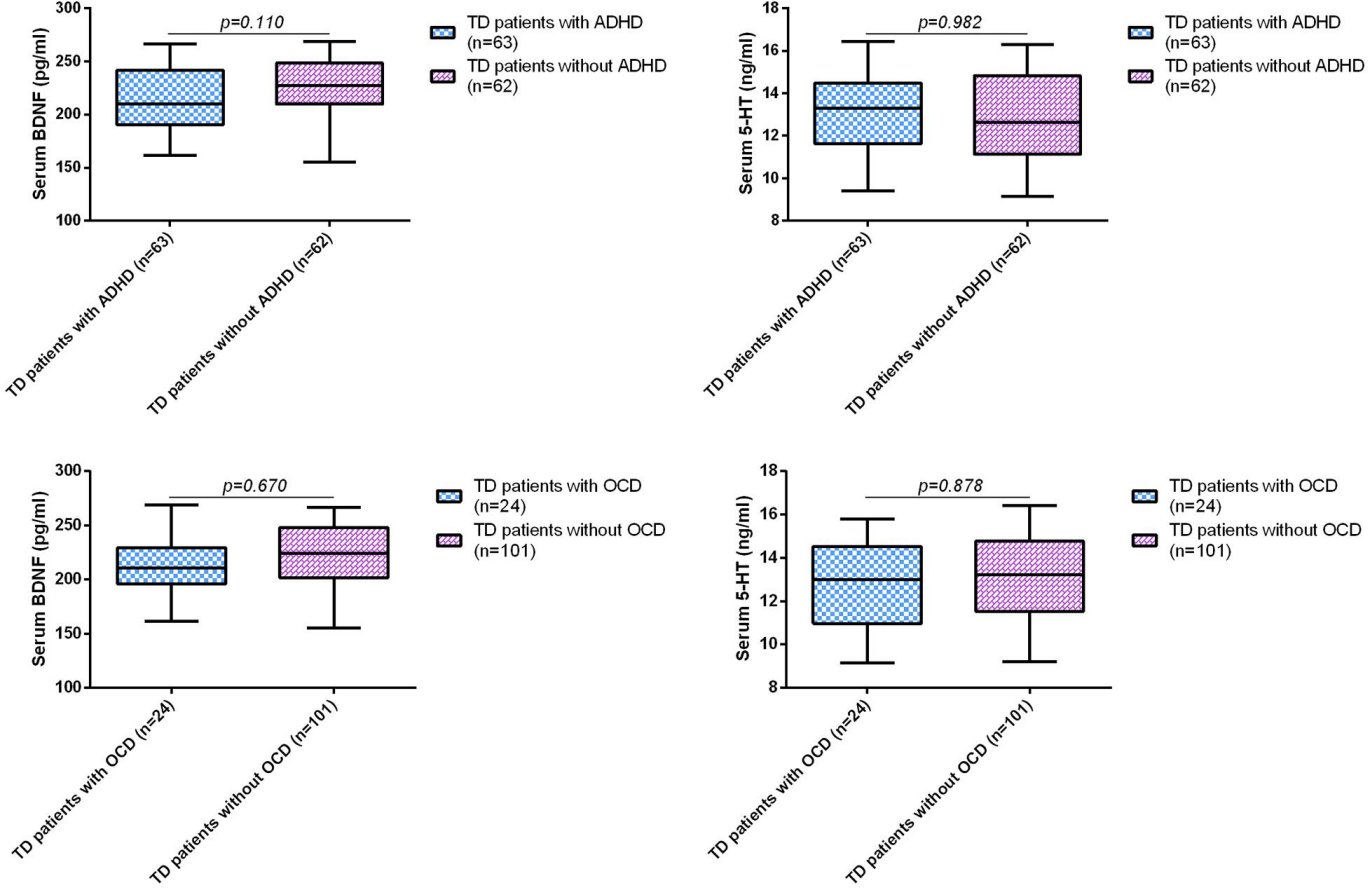
Serum BDNF and 5-HT levels in TD patients with or without comorbid ADHD and with or without comorbid OCD. BDNF, brain-derived neurotrophic factor; 5-HT, serotonin; TD, tic disorders; ADHD, attention-deficit/hyperactivity disorder; OCD, obsessive-compulsive disorder.

In the subgroup analysis of TD patients, serum BDNF and 5-HT levels were compared between children with and without comorbid ADHD or OCD. In this cohort, there were no significant differences in either biomarker between TD patients with ADHD and those without ADHD (Figure 4, BDNF: *p* = 0.110; 5-HT: *p* = 0.982). Similarly, no significant differences were observed between TD patients with OCD and those without OCD (BDNF: *p* = 0.670; 5-HT: *p* = 0.878).

### ROC analysis of serum BDNF and 5-HT for identifying TD and evaluating disease severity

**Figure 5:**
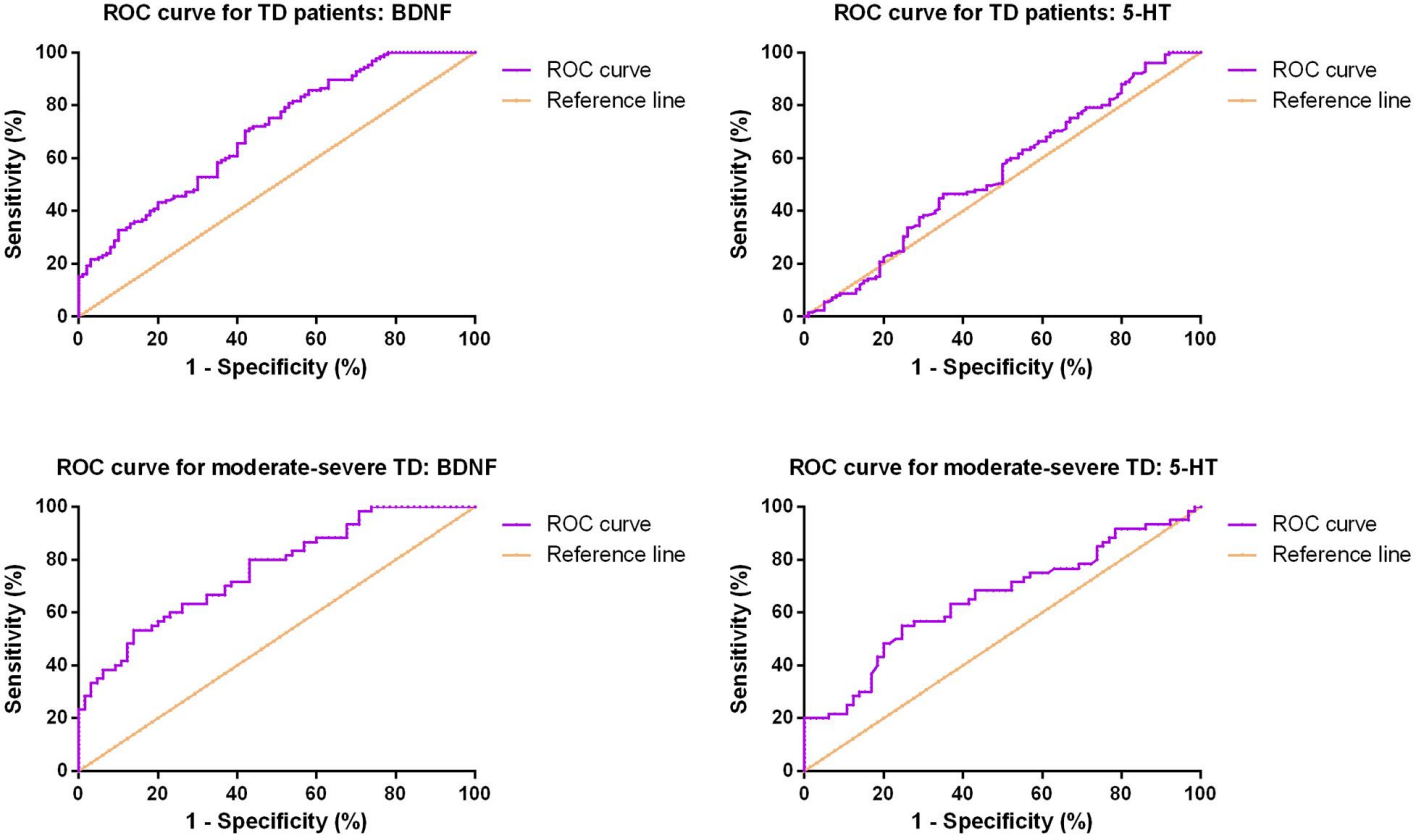
ROC curves of serum BDNF and 5-HT for the diagnosis of TD and for distinguishing moderate-to-severe TD from mild cases. ROC, receiver operating characteristic; BDNF, brain-derived neurotrophic factor; 5-HT, serotonin; TD, tic disorders.

In the ROC curve analysis, serum BDNF showed modest discriminative ability for identifying children with TD. The AUC for BDNF in diagnosing TD was 0.705 (95% CI 0.637–0.772), with an optimal cutoff value of 239.22 pg/mL, yielding a sensitivity of 59.0% and a specificity of 70.4%. In contrast, serum 5-HT showed limited discriminative ability for differentiating TD patients from healthy controls (*p* > 0.05). When distinguishing moderate-to-severe TD from mild cases, serum BDNF and 5-HT showed modest ability to distinguish moderate-to-severe TD from mild cases. The AUC for BDNF was 0.760 (95% CI 0.678–0.842), with a cutoff value of 216.86 pg/mL (sensitivity=73.8%, specificity=63.3%), while the AUC for 5-HT was 0.652 (95% CI 0.555–0.749), with a cutoff value of 13.18 ng/mL (sensitivity=63.1%, specificity=63.3%) (Figure 5).

### Logistic regression analysis of factors associated with TD in children

Binary logistic regression analysis was conducted to identify factors associated with the presence of TD in children. Variables were selected for the multivariable logistic regression model based on clinical relevance, biological plausibility, and potential confounding effects. The final model included age, sex, BMI, premature birth, sleep disturbances, family history of TD, PLT, serum BDNF, and serum 5-HT. The Hosmer–Lemeshow goodness-of-fit test indicated acceptable model calibration (*χ*^2^ = 2.444, df = 8, *p* = 0.964). Multicollinearity assessment showed that all included variables had low VIF values ranging from 1.011 to 1.071, indicating no significant multicollinearity. The analysis revealed that sleep disturbances (Table 2, OR = 3.976, 95% CI: 2.065–7.656, *p* < 0.001), family history of TD (OR = 5.353, 95% CI: 1.582–18.107, *p* = 0.007), and serum BDNF levels (OR = 0.971, 95% CI: 0.959–0.982, *p* < 0.001) were independently associated with TD.

**Table 2:** Logistic regression analysis of factors associated with TD in children.

Variables	Wald	Odds ratio	95% CI	*P*
Age	0.789	1.032	0.963–1.106	0.374
Sex	0.449	1.235	0.666–2.291	0.503
BMI	0.001	1.001	0.870–1.151	0.992
Premature birth	2.449	2.564	0.798–8.239	0.114
Sleep disturbances	17.046	3.976	2.065–7.656	<0.001
Family history of TD	7.280	5.353	1.582–18.107	0.007
PLT	1.683	1.004	0.988–1.011	0.195
BDNF	24.463	0.971	0.959–0.982	<0.001
5-HT	1.546	0.907	0.777–1.058	0.214

## Discussion

The clinical impact of childhood TD varies substantially, as many children present with mild tics and limited functional impairment, whereas others experience difficulties in school performance, social participation, and family functioning, particularly when comorbid ADHD or OCD is present (20, 21). Given the substantial functional impact of TD, identifying objective biological indicators has important clinical value. In the present study, serum BDNF levels were significantly lower in children with TD than in healthy controls, and the decrease was more pronounced in moderate-to-severe cases. Additionally, serum 5-HT levels were significantly lower in moderate-to-severe TD compared with both mild cases and healthy controls. Both biomarkers showed weak negative correlations with Yale Global Tic Severity Scale scores, and logistic regression confirmed that decreased BDNF was independently associated with TD.

BDNF plays a crucial role in neuronal development, maintenance, and synaptic plasticity through tropomyosin receptor kinase B (TrkB) signaling, and its dysregulation has been implicated in multiple neuropsychiatric disorders (22). Consistent with this neurobiological framework, our study found that serum BDNF levels were significantly reduced in children with TD, particularly in those with moderate-to-severe symptoms. These findings are in line with neurophysiological evidence showing that patients with Tourette syndrome exhibit impaired long-term potentiation (LTP)- and long-term depression (LTD)-like plasticity in the primary motor cortex, which reflects reduced BDNF-mediated synaptic adaptability (23, 24). Moreover, experimental data suggested that altered BDNF expression within the striatum interacted with dopaminergic and inflammatory signaling, contributing to abnormal motor outputs characteristic of tic behaviors (25). Therefore, these findings provide a mechanistic background for understanding the association between BDNF-related pathways and tic behaviors. In the present study, lower serum BDNF levels were associated with greater tic severity in children with TD. However, because serum BDNF is a peripheral measure, this finding does not necessarily indicate parallel changes in striatal BDNF/TrkB signaling. The relationship between circulating BDNF and central neurotrophic activity remains indirect, particularly because BBB permeability for BDNF is limited and still debated.

Serotonergic dysfunction had long been recognized as a neurochemical substrate of tic disorders. Neuroimaging studies demonstrated abnormalities in 5-HT₂A receptor density and serotonin transporter binding in patients with Tourette syndrome, indicating that impaired serotonergic regulation might have interacted with dopaminergic hyperactivity within cortico-striatal circuits (26). Experimental studies further showed that enhancement of 5-HT signaling mitigated tic expression; in animal models, fluoxetine and Jian-pi-zhi-dong decoction, a traditional Chinese herbal formula mainly containing herbs such as Codonopsis pilosula, Poria cocos, Uncaria rhynchophylla, Gastrodia elata, and Glycyrrhiza uralensis, increased striatal 5-HT concentrations and reduced 5-HT₂C receptor expression, thereby alleviating stereotyped behaviors and anxiety symptoms (27). Moreover, modulation of the gut microbiota was found to promote peripheral 5-HT synthesis, and fecal microbiota transplantation increased serum 5-HT while improving tic behaviors in experimental models (28). Importantly, a pediatric study reported no significant difference in plasma 5-HT levels between children with TD and healthy controls, possibly due to compensatory increases in serotonin transporter activity that enhanced 5-HIAA metabolism (15). Consistent with that observation, our findings also showed no overall difference in serum 5-HT between mild TD patients and controls. However, after stratified analysis, moderate-to-severe TD cases exhibited significantly lower 5-HT levels than mild cases. This trend suggested that peripheral 5-HT was not an independent etiologic factor but rather an auxiliary biological signal reflecting disease severity stratification.

These findings may also be interpreted within a broader developmental biomarker framework. Recent evidence from a 2D:4D digit ratio study suggested that prenatal hormonal exposure may be associated with TD susceptibility, supporting the relevance of trait-based developmental markers in TD (7). In this context, serum BDNF and 5-HT should be viewed as peripheral indicators related to neuroplasticity and neurotransmitter regulation, rather than direct substitutes for central neurochemical activity. This multi-level perspective links prenatal influences, neurodevelopmental trajectory, peripheral biomarkers, and clinical tic phenotype.

In subgroup analyses, serum BDNF and 5-HT did not differ between TD children with ADHD or OCD and those without these comorbidities. This pattern aligned with the notion that, although ADHD and OCD were frequent in TD and contributed substantially to clinical burden, their presence did not necessarily translate into distinct peripheral biomarker profiles (5). Neuroimaging studies had reported that serotonergic abnormalities in Tourette syndrome were more pronounced in patients with OCD comorbidity, showing altered serotonin transporter binding and receptor distribution in central circuits (14). These findings reflected changes at the central level, whereas our study focused on peripheral measurements. The absence of corresponding differences in serum 5-HT among comorbidity subgroups therefore suggested that peripheral 5-HT might capture systemic rather than central serotonergic alterations, providing a complementary perspective on TD pathophysiology. Taken together, these subgroup findings suggest that the observed BDNF and 5-HT patterns were more apparent across tic severity strata than across ADHD or OCD comorbidity status in this cohort. However, the negative subgroup results should be interpreted cautiously, particularly for OCD, because the subgroup sample size may have limited the ability to detect modest biomarker differences. This distinction emphasized that serum markers should be interpreted as complementary indicators rather than direct equivalents of central neurochemical alterations.

From a clinical perspective, the present findings should be interpreted as exploratory rather than immediately diagnostic. Current diagnosis and severity grading of TD still rely primarily on clinical evaluation and standardized behavioral scales. Although serum BDNF and 5-HT showed statistically significant but weak associations with TD status or symptom severity, their ROC performance was modest, particularly for 5-HT. Therefore, these markers should not be considered standalone diagnostic tools at this stage. Instead, they may provide auxiliary biological information related to neurotrophic and serotonergic alterations in pediatric TD. Larger multicenter cohorts and external validation are needed before serum BDNF and 5-HT can be considered for routine clinical assessment or treatment monitoring. Future studies should also include children with functional tic-like behaviors or atypical tic presentations to determine whether these peripheral markers can provide additional information for differential clinical assessment.

Several limitations of this study should be acknowledged. First, the sample size was relatively modest and drawn from a single center, which might limit the generalizability of the findings. Second, the cross-sectional design precluded conclusions regarding causal relationships between serum biomarkers and disease progression. Third, peripheral measurements of BDNF and 5-HT may not fully reflect central neurochemical dynamics, and cerebrospinal or neuroimaging data were not available for correlation analysis. Fourth, the ELISA measurements had a certain degree of assay variability, particularly for BDNF, for which the reported intra-assay and inter-assay CVs were up to 15%. This may have affected the precision of serum biomarker measurements. In addition, although previous treatment history was recorded, the potential residual influence of prior treatment exposure on serum BDNF and 5-HT levels could not be completely excluded. Other potential influences, including diet, sleep, emotional state, and pubertal development, also could not be fully ruled out because Tanner stage was not assessed in this cohort. Finally, the study focused on two biomarkers, and other neurotrophic or monoaminergic factors may also contribute to tic pathophysiology. Future multicenter longitudinal studies incorporating multimodal assessments are warranted to validate these findings and further elucidate the biological relevance of serum BDNF and 5-HT in pediatric tic disorders.

## Conclusion

In summary, this study demonstrated that serum BDNF levels were significantly decreased in children with TD and showed a stepwise decline with increasing disease severity. Reduced BDNF was independently associated with TD, suggesting its potential relevance as a peripheral marker of neurobiological dysfunction. In contrast, serum 5-HT levels did not differ between TD patients and healthy controls but declined significantly in moderate-to-severe cases, indicating that 5-HT may be more closely related to disease stratification than to disease presence. Given the modest ROC performance, these findings should be interpreted as exploratory. Serum BDNF and 5-HT may provide auxiliary information for understanding disease severity in pediatric TD, but their clinical use as diagnostic or stratification biomarkers requires validation in larger multicenter cohorts.

## Data Availability

The raw data supporting the conclusions of this article will be made available by the authors, without undue reservation.
